# Branchial Cleft Cyst Harbors Metastatic Papillary Thyroid Carcinoma

**DOI:** 10.7759/cureus.13940

**Published:** 2021-03-17

**Authors:** Dario A Marotta, Timothy J Morley, Maxwell J Jabaay, Leah O Grcevich, Ryan Gegg

**Affiliations:** 1 Department of Research, Alabama College of Osteopathic Medicine, Dothan, USA; 2 Department of Neurology, Division of Neuropsychology, University of Alabama, Birmingham, USA; 3 Department of Otolaryngology, Ear, Nose and Throat (ENT) South, Dothan, USA

**Keywords:** branchial cleft cyst, papillary thyroid carcinoma, metastatic cancer

## Abstract

Branchial cleft cysts are congenital anomalies which develop in utero, most commonly arising from the second branchial cleft. They are often asymptomatic lateral neck masses but can enlarge and become symptomatic in the setting of infection. The cystic cavity can form a potential space which can harbor infection and, in rare cases, malignant spread of primary tumors. Herein, we present a rare case of a 28-year-old male with an enlarging branchial cleft cyst of six months duration following an upper respiratory infection. Routine post-surgical histopathological examination of the excised mass revealed metastatic papillary thyroid carcinoma. To our knowledge, this case is one of five cases reported within the primary literature. This case draws attention to the occult nature of papillary thyroid carcinoma and the importance of routine histopathological examination of seemingly benign surgically excised lesions.

## Introduction

Branchial cleft cysts are the most common congenital neck masses arising laterally [1]. Approximately 95% of brachial cleft cysts arise from the second branchial cleft and occur anterior to the mid-sternocleidomastoid [2,3]. The majority of branchial cleft cysts are benign [1,4]. Many are discovered incidentally, remain asymptomatic, and are excised as a matter of cosmesis. In rare instances, they can become tender and rapidly enlarge following an upper respiratory infection (URI) [4]. Branchial cleft cysts can contain malignant tissue including papillary thyroid carcinoma (PTC) [5]. While the overall survival of PTC is as high as 90% when identified promptly, a delay in identifying metastases - such as those harbored within a purportedly benign branchial cleft cyst - are associated with reduced outcomes [6,7]. Herein, we report an illustrative case of metastatic papillary carcinoma within a congenital branchial cleft cyst. Post-surgical histopathological examination of the seemingly benign congenital cyst led to the discovery and treatment of the primary thyroid lesion. This case report serves to highlight this unique and underreported dilemma.

## Case presentation

**Figure 1:**
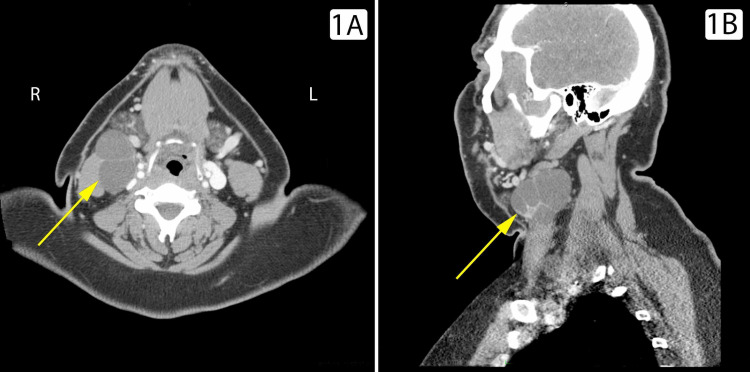
Computed tomography of the head and neck. Axial (A) and sagittal (B) computed tomography (CT) imaging revealing a 6.5 cm x 4 cm cystic mass (yellow arrow) at level 3, consistent with a second branchial cleft cyst.

A 28-year-old male was referred to the otolaryngology clinic for a mildly tender lateral neck mass that first appeared six months prior to presentation. The mass steadily grew in size. It was initially accompanied by mild sinus congestion without constitutional symptoms, dysphagia, dysphonia, shortness of breath, or any other unusual symptoms. Aside from a body mass index of 49, the patient had no pertinent medical history. He reported a 10-pack-year smoking history and social alcohol consumption limited to three to four beers weekly. His family history was significant for unspecified lung malignancy. Physical examination revealed a 5-cm cervical mass inferior to the mandible on the lateral neck. There was no stridor, the trachea was midline, and the thyroid was non-tender without palpable nodules. No other masses were appreciated on palpation of the neck. Contrast-enhanced computed tomography (CT) of the head and neck revealed a 6.5 cm x 4 cm cystic mass at level 3, consistent with a second branchial cleft cyst (Figures 1A, 1B).

**Figure 2:**
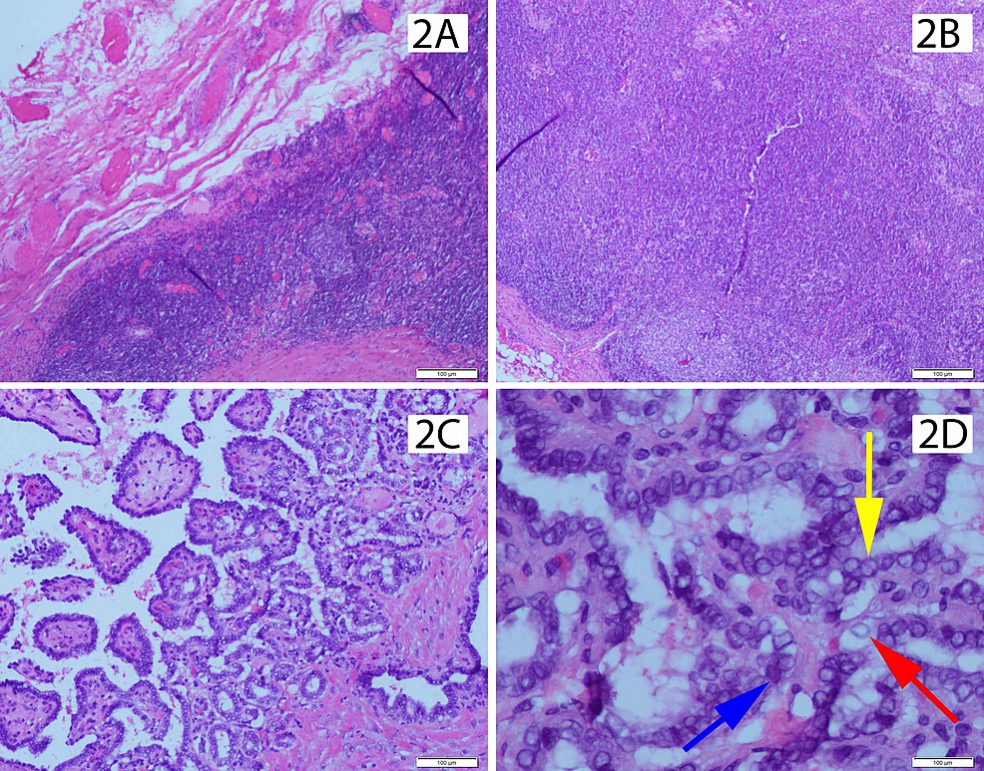
Histopathological examination of excised surgical specimen. (A) Squamous and ciliated respiratory epithelial consistent with branchial cleft cyst wall. (B) Lymphoid tissue consistent with branchial cleft cyst. (C) Papillary structures with loose fibrovascular cores. (D) Nuclear atypia with crowding, hyperchromatic nuclei (blue arrow), nuclear grooves (yellow arrow), and chromatin clearing with margination and glass nuclei (red arrow).

Fine needle aspiration of the cystic content was benign and without malignant cells. The branchial cleft cyst was surgically excised under general anesthesia without complications and the excised specimen was forwarded to pathology for further evaluation. Histopathological examination revealed a cystic component, delineated by squamous and ciliated respiratory epithelium, containing papillary structures with loose fibrovascular cores and hyperchromatic nuclei consistent with classic PTC (Figures 2A-2D).

Immunohistochemical staining was cytokeratin 7 (CK 7) positive and thyroid transcription factor-1 (TTF-1) positive, commensurate with a diagnosis of metastatic PTC. Subsequent ultrasound of the thyroid revealed a 1.5-cm solid isoechoic nodule in the right lobe with characteristics correlating with a thyroid imaging reporting and data system (TI-RADS) 5 lesion. Fine need aspiration of the contents revealed thyroid follicle cells showing features suspicious for papillary carcinoma.

The patient underwent a total thyroidectomy and was noted to have three separate foci of well-differentiated PTC in the right lobe with a small micro-carcinoma focus within the left lobe. Enlarged lymphadenopathy discovered in level 6 of the neck was identified as a site of metastases with extension outside of the lymph node into the soft tissue. Other foci were contained within the surgical margins. Following thyroidectomy, the patient was treated by a multi-disciplinary care team consisting of representatives from otolaryngology, endocrinology, and radiation oncology. At the patient’s six-week follow-up appointment, the patient had no particular neck complaints. The patient elected for aggressive radioactive iodine ablation and long-term thyroid suppression.

## Discussion

Branchial cleft cysts are embryological remnants that fail to close in utero [1,3]. These cysts are typically lined with squamous epithelium and can form a potential space when inflamed. While branchial cleft cysts often remain asymptomatic, episodic swelling can occur. On occasion, URIs can induce rapid growth and increase the risk for secondary infection and airway obstruction [1,4]. These triggering symptoms can prompt a patient to seek medical attention, such as in this case. The primary treatment for symptomatic branchial cyst is surgical excision following diagnostic evaluation with CT and ultrasound [8]. Histopathological and immunohistochemical analyses of post-surgical specimens are common practice in the United States. Most branchial cleft cysts are benign; however, a modicum of specimens harbors metastatic components. In a small retrospective study, three of 28 branchial cleft cysts observed over eight years at a single institution contained metastatic tissue; 66% were PTC and 33% were tonsillar squamous cell carcinoma [5].

PTCs are common thyroid tumors with metastatic potential. These tumors share an occult clinical picture consistent with asymptomatic congenital neck masses [8]. Many instances are non-palpable “incidentalomas” evading routine thyroid examinations only to be detected serendipitously through unintentional imaging. In fact, autopsy studies have shown 30%-60% of patients have non-palpable thyroid masses; the majority (87%) are without malignant potential and can be safely monitored with physical exam and routine imaging alone [9]. Rapidly enlarging head and neck masses, masses with a diameter greater than 1 cm, vocal cord paralysis, hoarseness, cervical lymphadenopathy, excessive childhood exposure to radiation, and family histories pertinent for familial cancers are suspect for malignancy [10]. Notwithstanding, overall survival is as high as 90% at 10 years with prompt treatment initiation [7]. Age at diagnosis and delays in identifying metastatic spread are both associated with poorer overall outcomes [6].

PTC metastasis within the confines of a branchial cleft cyst has been reported in the literature, albeit infrequently [11-14]. A review of these cases revealed a similar set of circumstances. That is, the patient underwent excision of a branchial cleft cyst with unexpected histological staining patterns consistent with PTC. Radiologic imaging was generally benign with purported post-hoc evidence suspicious of nonconformity in only one case. Atypical characteristics for second branchial cysts, such as wall thickening, enhancement, dystrophic calcifications, and development of septations, may hint at complex branchial cleft cyst pathology [11]. Even with these findings, the overlap between other neoplastic, infectious, and inflammatory etiologies may still require excision with post-excisional tissue analysis. In each of the aforementioned cases, tissue analysis led to the subsequent identification of primary thyroid lesions in the absence of discernable signs and symptoms.

Fortunately, in this case, the patient sought care when the branchial cleft cyst became symptomatic following an upper respiratory illness. In the absence of symptoms, the patient would have been unlikely to seek medical treatment. This patient had limited financial resources and time away from work which hindered his ability to seek routine medical care. Further, the patient was morbidly obese with a body mass index of 49, thereby obfuscating thyroid testing and limiting detection even with routine thyroid physical examination [15]. Taken together, diagnosis and treatment of the occult malignancy in this patient would have likely been delayed without his triggering symptoms. Patients of similar socioeconomic and health status may be at risk given that branchial cleft cyst excision is considered elective in the absence of symptoms and delayed diagnoses can lead to poorer outcomes. The paucity of data available for patients with branchial cleft cysts, particularly those with concomitant metastatic involvement, creates a barrier for future research. As more cases are discovered and reported, metanalyses may serve to uncover more details regarding this rare association.

## Conclusions

Metastatic dissemination of PTC within the confines of a second branchial cleft cyst is a rare finding with only four other cases previously documented in the literature. This case report serves to illustrate the importance of routine pathological examination of surgical specimens, even in those lesions of seemingly benign etiology. While PTC is amenable to treatment if identified early, harboring of a metastatic lesion within a benign congenital mass may lead to delayed treatment and reduced overall survival. More research is needed to discern the impact of socioeconomic status in concert with this unique clinical scenario.
